# Nerve Block for Septorhinoplasty: A Retrospective Observational Study of Postoperative Complications in 24 Hours

**DOI:** 10.7759/cureus.6961

**Published:** 2020-02-12

**Authors:** Mohammed Elsayed, Razan A Alosaimy, Nujod Y Ali, Mohammad A Alshareef, Ahmed H Althqafi, Mohannad K Rajab, Abdullah S Assalem, Ahmed J Khiyami

**Affiliations:** 1 Otolaryngology Head and Neck Surgery, King Abdullah Medical City, Makkah, SAU; 2 Otolaryngology Head and Neck Surgery, Umm Al-Qura University, Makkah, SAU; 3 Otolaryngology Head and Neck Surgery, King Abdulaziz Medical City, Jeddah, SAU; 4 Otolaryngology Head and Neck Surgery, King Fahad General Hospital, Jeddah, SAU; 5 Otolaryngology Head and Neck Surgery, Armed Force Hospital, Ministry of Defense, Taif, SAU

**Keywords:** septorhinoplasty, nerve block, local analgesia., infraorbital, rhinoplasty

## Abstract

Septorhinoplasty is a surgical procedure that provides functional improvements and esthetic adjustments to the appearance of the nose. Pain is a common postoperative complication, and pain management is known to decrease postoperative complications and total cost. Local anesthetics can cost-effectively decrease postoperative pain scores and reduce analgesic requirements.

The primary objective of this study was to assess the effect of bilateral facial nerve blocks given with general anesthesia on pain scores and the use of postoperative analgesia. The secondary objective was to compare the vital signs stability between a group given bilateral facial nerve blocks with general anesthesia and a group given general anesthesia only.

We conducted a retrospective observational study among 40 patients who were divided into two groups, each containing 20 patients. The patients in the nerve block (NB) group received general anesthesia and bilateral facial blocks of the infraorbital and infratrochlear nerves via 5 ml of 0.25% levobupivacaine with 5 ml of diluted adrenaline 1:100,000. Patients in the Control group received general anesthesia only. Both groups received the same local injection of a mixture of 5 ml of 1% lidocaine and 5 ml of 1:100,000 epinephrine at the surgical site, along with the standard general anesthesia. A numerical rating scale, the visual analog scale (VAS), was used to evaluate postoperative pain at 15, 30, and 45 minutes postoperatively, and the stability of the vital signs was also assessed.

The results showed that using bilateral infraorbital and infratrochlear nerve block injection with 0.25% levobupivacaine for patients who underwent septorhinoplasty under general anesthesia provided greater stability of vital signs but had no effect on the pain score or analgesia need. Further assessment should be performed in a larger number of patients to either confirm or refute these results. Additional studies could be conducted in several hospitals within the Kingdom to determine how broadly applicable nerve blockade is in reducing pain sensation.

## Introduction

Septorhinoplasty is a surgical procedure for aesthetically enhancing the appearance of the nose and repairing nasal deformities. The most common postoperative complications are pain, edema, and periorbital ecchymosis. Forty-five percent of patients have reported pain scores > 3 on a 0 to 10 numerical rating scale (NRS), and approximately 30% have reported pain scores of 6 [1-2]. Postoperative pain therapy is known to decrease postoperative complications and total cost [3]. Some clinical guidelines recommend providing postoperative pain therapy, such as those of the American Society of Anesthesiologists (ASA) who recommend the use of analgesic and regional nerve blockade strategies whenever possible [4-5]. The use of local anesthesia is a cost-effective option for decreasing postoperative pain scores and reducing analgesic requirements, and it can be used in plastic surgery as a primary option procedure [6]. Local analgesic techniques are beneficial adjuncts to an anesthetic protocol. Along with general anesthesia, bilateral infraorbital nerve blocks have been shown to have beneficial effects on pain management following nasal surgery in a day surgery unit because the blocks supply a major area of the nose [7-8]. The infraorbital nerve blocks can be used either with an intraoral or extraoral technique [9]. To date, there has been insufficient data to show a difference between intraoral or extraoral nerve blocks in nasal surgery [10]. Peripheral nerve blocks work by injecting a local anesthetic, such as levobupivacaine, around the nerve that supplies the surgical area. By changing the permeability of cell membranes to sodium, local anesthetics can prevent the transmission of nerve impulses [11]. In a study conducted in 2007 that compared bilateral infraorbital nerve block with intravenous (IV) fentanyl for analgesia following cleft lip repair in children, a bilateral infraorbital block was found to have a better analgesic effect, reduced time to awakening, and improved appetite relative to those of fentanyl [12]. In a 2014 study that compared local vs. systemic analgesia for post-thoracotomy management in infants and evaluated the safety and effect of a local nerve block found that local analgesia was effective and decreased postoperative care [13]. Numerous drugs are used in a nerve blockade. As shown in a study that investigated the duration of action of bupivacaine, levobupivacaine, ropivacaine, and pethidine in peripheral nerve block in the rat, equal doses of the investigated local anesthetics exerted similar durations of sensory blockage in a peripheral nerve block model in rats [14]. Septorhinoplasty involves surgical sites that are innervated by the infratrochlear nerve, a terminal branch of the ophthalmic nerve that provides sensation to the skin of the dorsum of the upper part and both sides of the nose. However, infratrochlear nerve blocks have been poorly investigated for this indication [9, 15]. Peripheral nerve blocks cause fewer side effects and complications, such as less edema in the surgical site and less pain. Infraorbital and infratrochlear nerve blocks are simple and easy to use with few complications, provide analgesia after surgery, reduce complications related to anesthesia during the recovery period, avoid the use of perioperative opioids, provide excellent pain control, and reduce inhalant and injectable anesthetic requirements. Therefore, peripheral nerve blocks are used currently as part of a multiple analgesic strategy to provide safe and effective postoperative pain therapy with minimal side effects.

The primary objective of this study was to assess the effect of bilateral facial nerve blocks given with general anesthesia on pain scores and the use of postoperative analgesia. The secondary objective was to compare the vital signs stability between a group given bilateral facial nerve blocks with general anesthesia and a group given general anesthesia only.

We hypothesized that the use of bilateral extraoral infraorbital and infratrochlear nerve blocks with 0.25% levobupivacaine for patients who undergo septorhinoplasty under general anesthesia would reduce the needed dose of postoperative analgesia, reduce the pain score, and enhance the stability of the vital signs. The null hypothesis was that there would be no reduction in the need for postoperative analgesia, pain score, or vital stability after administration of 0.25% levobupivacaine as a nerve blocker.

## Materials and methods

Study design

This was a single-center retrospective study conducted at King Abdullah Medical City (KAMC), Makkah, Saudi Arabia. The study was conducted between January 2017 and January 2018 and was approved by the ethical committee of KAMC. The population included all patients in the Otolaryngology Clinic at KAMC. Informed written consent was provided by all participants in this study. The inclusion criteria were all patients with an indication for septorhinoplasty. The exclusion criteria were age < 18 years old, patients with hypertension and ischemic heart disease, patients with BMI > 30, patients with preoperative chronic pain, and patients with an American Society of Anesthesiologists (ASA) classification III-IV. After applying the inclusion and exclusion criteria, only 40 adult patients were selected for the study. The patients were divided randomly into two groups of 20 patients each. The patients in the nerve block (NB) group received general anesthesia in addition to bilateral facial blocks via 5 ml of 0.25% levobupivacaine with 5 ml of diluted adrenaline 1:100,000. The patients in the Control group received general anesthesia only.

Anesthetic, nerve block, and surgical techniques

Both groups received the same standard general anesthesia. In the NB group only, following the tracheal intubation, the primary surgeon performed the bilateral facial block, consisting of infraorbital and infratrochlear blocks. The infraorbital nerve block was performed by using an extraoral approach by palpating the infraorbital ridge to locate the infraorbital foramen. Then, a 25-gauge needle was inserted laterally to the nostril and advanced forward 1-2 cm until it was felt beneath the finger locating the foramen. Next, with great caution not to enter the foramen itself and after negative aspiration of blood, 4 ml of 0.25% levobupivacaine with 4 ml of diluted adrenaline 1:100,000 were injected. The infratrochlear injection was made by inserting the needle 1 cm above the inner canthus and then injecting 1 ml of 0.25% levobupivacaine with 1 ml of diluted adrenaline 1:100,000. All of the patients (40) received 10 ml of local injection in the form of a mixture of 5 ml of 1% lidocaine and 5 ml of 1:100,000 adrenaline that was injected into the septum, the subcutaneous area at the dorsum, the tip of the nose, the site of the marginal incision and columellar incision, and the sites of the osteotomies. All patients underwent open septorhinoplasty by the same primary surgeon. The procedure involved lateral and medial osteotomies and cartilage grafts. Standard analgesia was given to both groups before the end of the surgery.

Patient assessment

After extubation, all of the patients were moved to the recovery area for observation where they were evaluated for postoperative pain at 15, 30, and 45 min after the surgery by using the visual analog scale (VAS) varying from 0 (no pain) to 10 (maximum). For patients with a VAS score > 3, IV morphine was given. We assessed the vital sign stability of the patients postoperatively by measuring heart rate and mean arterial blood pressure (MAP) and comparing them with normal values, then categorizing them into two groups: vitally stable or vitally unstable. Every patient was assessed for vital sign stability once before discharge after recovery. The patients were moved to the daycare unit and evaluated for postoperative complications (nausea and vomiting, facial hematoma, somnolence, and edema). After discharge, the patients were asked to return to the clinic 24 hours later for evaluation.

Statistical analysis

The demographic data of the two groups were compared. A value of p < 0.05 was considered to be indicative of statistical significance. The statistical analysis was performed by using the Chi-squared test and binomial test in the IBM Statistical Package for Social Sciences (SPSS) software (IBM SPSS Statistics, Armonk, NY). The Chi-squared test was used to compare a group’s values to hypothetical values (e.g., vital signs and analgesia) and the binomial test was used to compare values (e.g., pain score and age) in two unpaired groups.

## Results

**Figure 1:**
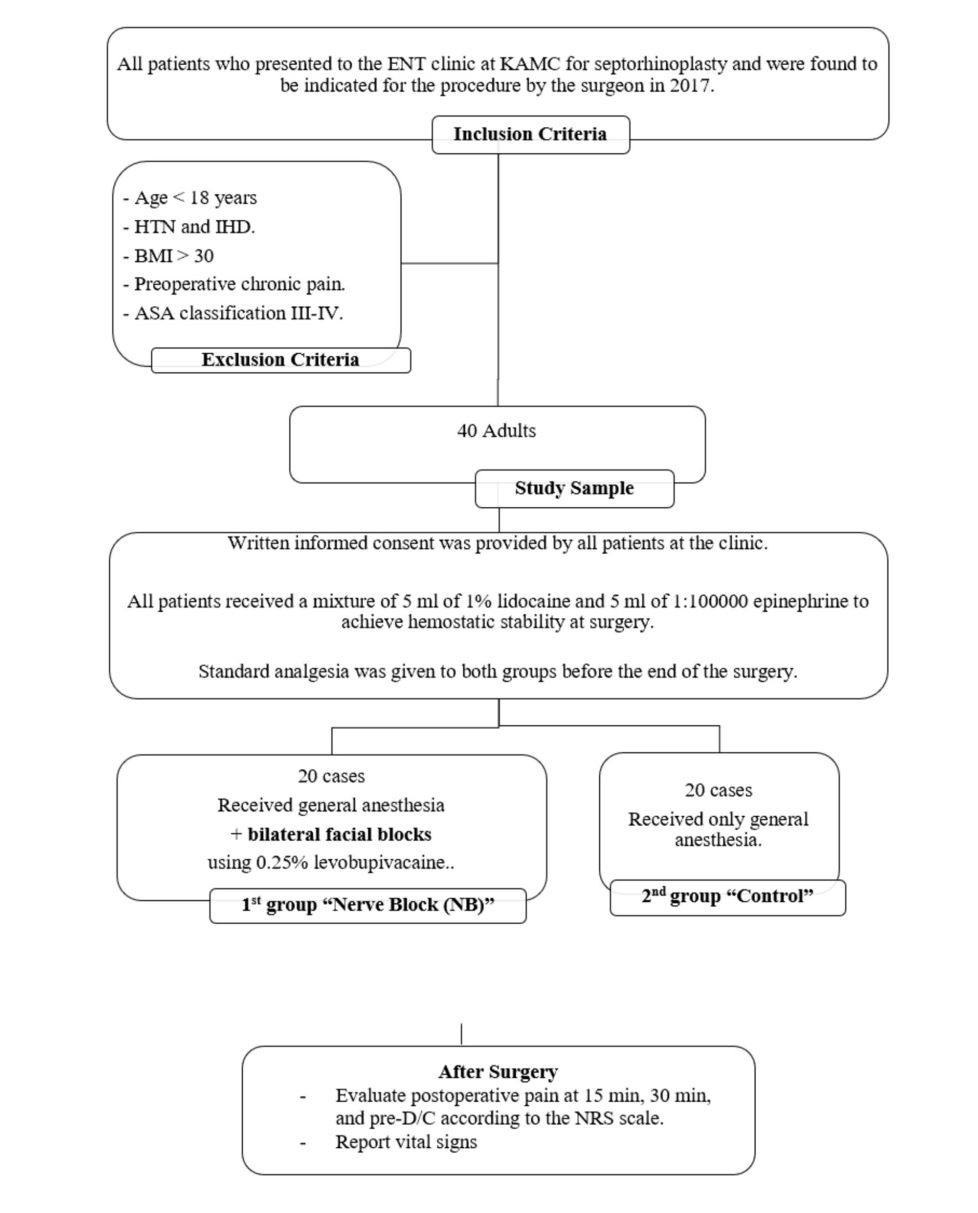
The study flow chart ASA: American Society of Anesthesiologists; BMI: body mass index; D/C: discharge; HTN: hypertension; IHD: ischemic heart disease; KAMC: King Abdullah Medical City; NRS: numeric rating scale

Forty patients (26 male and 14 females) were included from January 2017 to January 2018 for septorhinoplasty. The study flow chart is presented in Figure 1.

The morphometric characteristics were similar between groups (Table 1). From the data shown in Table 1, the sample contained more male participants than females (65% and 35%, respectively).

**Table 1:** Demography of the Patients in the Nerve Block (NB) and Control Groups

	NB (n = 20)	Control (n = 20)	P-value
Age (years)	26 (8)	28 (9)	0.416
Male	15 (75%)	11 (55%)	0.133
Female	5 (25%)	9 (45%)	

Regarding the type of operation, 85% of the participants in the NB group had a primary septorhinoplasty operation, 10% had septorhinoplasty as revision surgery, and 5% had septorhinoplasty with sixth and seventh rib grafts (Table 2). In the Control group, 95% of the participants underwent primary septorhinoplasty, whereas only 5% underwent septorhinoplasty as revision surgery. To understand the effect of NB on the pain score, vital signs stability, and postoperative analgesia, every point was analyzed separately.

**Table 2:** Type of Operation NB: nerve block; SRP: septorhinoplasty

		NB (n = 20)	Control (n = 20)
Primary SRP		17 (85%)	19 (95%)
SRP revision		2 (10%)	1 (5%)
SRP with 6^th^ and 7^th^ rib grafts		1 (5%)	0

The VAS scores for pain (mean (standard deviation)) were lower after 15 minutes in the Control group (0.7 (0.7)) than in the NB group (1.1 (1.7), P = 0.813) and after 30 minutes (0.5 (0.6)) than in the NB group (0.6 (0.7), P = 0.774). Moreover, at pre-discharge, the NRS score was lower in the Control group (0.3 (0.6)) than in the NB group (0.6 (0.6), P = 0.162) (Table 3).

**Table 3:** Postoperative Pain Score in the Nerve Block (NB) and Control Groups After 15 Minute, 30 Minute, and 45 Minute Intervals D/C: discharge; VAS: visual analog scale

	NB group							Control group						
Pain score	1	2	3	4	5	6	No pain	1	2	3	4	5	6	No pain
VAS 15 min														
No. of patients	6	2	0	0	1	1	10	7	4	0	0	0	0	9
% of patients	30%	10%			5%	5%	50%	35%	20%					45%
VAS 30 min														
No. of patients	6	2	0	0	0	0	12	8	1	0	0	0	0	11
% of patients	30%	10%					60%	40%	5%					55%
VAS 45 min (Pre-D/C)														
No. of patients	8	1	0	0	0	0	11	4	1	0	0	0	0	15
% of patients	40%	5%					55%	20%	5%					65%

Regarding the primary outcome, pain was assessed postoperatively at three different time points, and no statistically significant differences in pain were observed (Table 4).

**Table 4:** Distribution of the Means, Standard Deviations, and P-values in the Nerve Block and Control Groups BP: blood pressure; D/C: discharge; HR: heart rate; VAS: visual analog scale

	NB (n = 20)	Control (n = 20)	P-value
VAS 15 min	1.1 (1.7)	0.7 (0.7)	0.813
VAS 30 min	0.6 (0.7)	0.5 (0.6)	0.774
VAS 45 min (Pre-D/C)	0.6 (0.6)	0.3 (0.6)	0.162
HR	80 (7)	79 (15)	0.303
Systolic BP	108 (10)	110 (13)	0.607
Diastolic BP	64 (7)	63 (10)	0.533
Mean arterial pressure (MAP)	79 (7)	78 (10)	
Vitally unstable	2 (18%)	9 (82%)	0.018 (< 0.05)
Postoperative analgesia administration	2 (10%)	0	0.137

There were no significant differences in the vital signs (blood pressure and heart rate) after surgery. However, the NB group showed significantly more stable vital signs than those in the Control group (P = 0.018) (assessed by measuring the MAP and HR and comparing them with normal values).

Finally, there was no significant difference in the need for postoperative analgesia administered, which was needed only for two patients in the NB group (P = 0.137). The first patient received 3 mg of morphine, whereas the other patient received 50 μg of fentanyl and 1 mg of midazolam. All results are presented in Table 4. There were no protocol deviations or adverse events related to the study procedures.

## Discussion

Effective treatment of postoperative pain should facilitate early mobilization, fluid and food intake, and the resumption of normal physical activities [16]. However, effective analgesic treatment remains a challenge. Postoperative pain affects many patients worldwide and the postoperative period includes a high risk of morbidity and mortality, some of which may be related to analgesics [17-19].

The surgical patient is often treated with a combination of non-opioid analgesics, referred to as “multimodal analgesia” [16]. The aim of this strategy is to achieve a synergistic or additive beneficial effect with the lowest doses of each analgesic, thus preventing harmful effects while decreasing the use of opioids and consequently, opioid-related adverse events [20-21]. The most commonly used non-opioids in multimodal analgesic treatment are paracetamol, non-steroidal anti-inflammatory drugs (NSAIDs), steroids, ketamine, local anesthetics, and gabapentinoids [22]. Currently, many different combinations of non-opioid analgesics are employed in clinical practice [20, 23]. However, the knowledge regarding risks, potential additive or synergistic analgesic effects, and the individual patient response related to such combinations is insufficient [21, 24].

A prospective clinical study compared postoperative pain in patients undergoing certain otorhinolaryngologic procedures under local anesthesia with sedation and found that pain was significantly higher in patients undergoing septorhinoplasty compared with septoplasty [25]. These findings are similar to the ones found by another study where patients undergoing septoplasty did not require the use of postoperative analgesia and remained within the acceptable pain range of analgesic success throughout the postoperative period studied [26]. Within the same study, patients who were undergoing surgical correction of the whole nose reported a gradual decline in pain, which reached a similar level to the pain observed in the post-septoplasty patients only six days following the surgical procedure. This is why a method to reduce the pain post-septorhinoplasty, especially during the first few days post-surgery, is required

The use of bilateral nerve blocks during nasal surgery has been reported in the literature. A study done by Boselli et al. demonstrated that bilateral facial blocks using an extraoral approach and infra-trochlear blocks reduced the use of morphine significantly postoperatively [27]. Furthermore, the same study showed that using that method also showed a reduction in time spent in the intensive care unit and after operative hospitalization.

In contrast to previous studies that found that 45% of patients reported pain scores > 3 on the 0 - 10 NRS [1-2], our study found that only 5% of the patients had pain scores > 3. Previous studies have found good effects on postoperative pain management from the use of bilateral infraorbital NBs [28], but our results showed that the effects of these NBs on reducing postoperative pain were not significant after 15 minutes, 30 minutes, and 45 minutes (P = 0.813, 0.774, and 0.614, respectively). In addition, there was no significant difference in the need for postoperative analgesia administration (P = 0.137), which was needed only for two patients in the NB group.

In this study, in addition to investigating the effectiveness of NBs on reducing postoperative pain and the need for analgesia, we also assessed their effect on the postoperative stability of the vital signs. We found that the use of 0.25% levobupivacaine as an NB significantly (P = 0.018) improved vital stability and reduced the incidence of vital instability. However, bilateral infraorbital and infratrochlear nerve blocks with 0.25% levobupivacaine require technical expertise from a variety of clinicians. Nurses also must be experienced in preoperative screening and assessment of this patient population during the intraoperative and postoperative phases. They must observe patients for potential side effects and complications to assess the levels of nerve blocks and the quality of analgesia that may not be suitable for some patients.

There were some limitations to this study that should be considered. This was a single-center, retrospective, observational method with a small sample size (40 patients). Further studies conducted as multicentric randomized controlled trials would give greater confidence in the results.

## Conclusions

The use of bilateral extraoral infraorbital and infratrochlear nerve blocks with 0.25% levobupivacaine for patients undergoing septorhinoplasty under general anesthesia was found to enhance vital stability but did not reduce postoperative pain or the need for postoperative analgesia. Expert medical teams should be used for the injection of nerve blocks. Further studies should be conducted in a higher number of patients to obtain more representative and accurate results. We recommend that researchers conduct similar studies in hospitals within the Kingdom to help surgeons understand the effects of nerve blockade on postoperative pain, the need for analgesia, and vital sign stability.
